# Associations of Multimarkers of Metabolic Malnutrition and Inflammation With All‐Cause Mortality and Their Interplay With Thyroid Function

**DOI:** 10.1002/edm2.70162

**Published:** 2026-02-06

**Authors:** Setor K. Kunutsor, Reyhaneh Rikhtehgaran, Yanning Xu, Margery A. Connelly, Irina Shalaurova, Layal Chaker, Stephan J. L. Bakker, Robin P. F. Dullaart

**Affiliations:** ^1^ Section of Cardiology, Department of Internal Medicine, Rady Faculty of Health Sciences University of Manitoba Winnipeg Manitoba Canada; ^2^ Department of Internal Medicine, Academic Center for Thyroid Diseases Erasmus University Medical Center Rotterdam the Netherlands; ^3^ Department of Epidemiology Erasmus University Medical Center Rotterdam the Netherlands; ^4^ Labcorp Morrisville North Carolina USA; ^5^ Department of Internal Medicine, Division of Nephrology, University Medical Center Groningen University of Groningen Groningen the Netherlands; ^6^ Department of Internal Medicine, Division of Endocrinology, University Medical Center Groningen University of Groningen Groningen the Netherlands

**Keywords:** autoimmunity, cohort study, inflammation, malnutrition, metabolic vulnerability index, mortality, thyroid function

## Abstract

**Introduction:**

The metabolic vulnerability index (MVX)—a composite biomarker reflecting metabolic malnutrition and inflammation—has been linked to increased mortality risk in populations with cardiovascular disease. Thyroid function, a key regulator of metabolism and inflammation, may confound or modify this relationship, but evidence in the general population is limited.

**Objectives:**

To evaluate the interplay between MVX and its subcomponents (inflammation vulnerability index, IVX and metabolic malnutrition index, MMX), thyroid function, and mortality risk in the general population.

**Methods:**

In the PREVEND prospective study, which included 5446 participants (mean age 54 years; 49.9% male), both MVX (estimated using six metabolites measured simultaneously through nuclear magnetic resonance spectroscopy) and thyroid function (FT3, FT4, TSH) were evaluated at baseline. Hazard ratios (HRs) with 95% confidence intervals (CIs) for all‐cause mortality were estimated.

**Results:**

During a median follow‐up of 14.1 years, 806 deaths were recorded. Spline analyses showed graded dose–response relationships of MVX, IVX and MMX with mortality risk. In separate analyses adjusted for several established risk factors, the HRs (95% CIs) of mortality were 1.28 (1.18–1.38), 1.23 (1.14–1.32) and 1.16 (1.07–1.25) per 1 standard deviation increment in MVX, IVX and MMX, respectively. The HRs remained consistent on further adjustment for FT3, FT4 and TSH. Sex as well as levels of FT3, FT4 and TSH did not significantly modify the associations.

**Conclusions:**

The MVX and its subcomponents (IVX and MMX) are independently associated with all‐cause mortality, consistent with graded dose–response relationships. Thyroid function does not confound or modify these associations.

## Graphical abstract

MVX, Thyroid Function and Mortality.

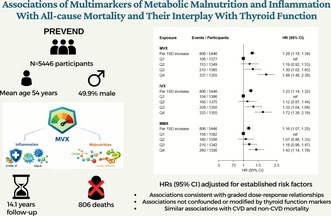

## 1 Introduction

In older adults and people with chronic illnesses—such as kidney or liver disease, heart failure, chronic obstructive pulmonary disease, rheumatoid arthritis, or cancer—a combination of malnutrition and inflammation is common (i.e., malnutrition‐inflammation syndrome) and has been linked to an excess mortality risk [1, 2]. In 2023, Otvos and colleagues used clinical nuclear magnetic resonance (NMR) analysis to quantify six metabolites in plasma plausibly linked to the metabolic malnutrition‐inflammation syndrome and derived the metabolic vulnerability index (MVX) ‐ a composite biomarker reflecting metabolic malnutrition and inflammation [3]. The MVX is designed to provide a robust measure of systemic metabolic vulnerability and has been shown to be associated with increased mortality risk in cardiac catheterisation patients or those presenting with heart failure [3, 4]. The MVX comprises two contributing multimarkers: the inflammation vulnerability index (IVX), which captures systemic inflammatory processes, and the metabolic malnutrition index (MMX), which reflects metabolic nutritional status.

Thyroid hormones (triiodothyronine (FT3), free thyroxine (FT4) and thyroid‐stimulating hormone (TSH)) play a central role in regulating metabolism and inflammation [5, 6] and are associated with the risk of adverse cardiovascular outcomes including mortality. For instance, a recent individual participant data meta‐analysis showed a J‐shaped association of FT4 with CVD and mortality, while low TSH levels were associated with cardiovascular and all‐cause mortality [7]. In severe chronic illness and states of malnutrition—particularly protein‐energy malnutrition—disruptions in thyroid function are common. These often manifest as low circulating levels of FT3, sometimes accompanied by reduced levels of FT4, without a compensatory rise in TSH. This pattern is characteristic of non‐thyroidal illness syndrome (NTIS) [8, 9]. NTIS has been linked to worse prognosis in patients with various comorbidities, including cardiovascular [10] and renal disease [11], suggesting it may contribute to or amplify underlying metabolic vulnerability. Given the biological plausibility and prior literature indicating links between NTIS, chronic disease burden, and mortality, we hypothesised that thyroid function might influence or confound the associations of MVX and its subcomponents with mortality risk. Whether thyroid function confounds or modifies the associations of MVX, IVX and MMX with mortality has not been previously investigated.

Additionally, thyroid autoimmunity, reflected by the presence of thyroid peroxidase antibodies (TPOAb), may influence cardiovascular and metabolic risk through shared mechanisms such as chronic inflammation, oxidative stress and endothelial dysfunction [12]. TPOAb positivity has been observed even in euthyroid individuals [13] and is associated with subclinical atherosclerosis and cardiometabolic abnormalities [14, 15], raising the possibility that thyroid autoimmunity could also modify the relationships between metabolic vulnerability and mortality. Using data from the population‐based Prevention of Renal and Vascular ENd‐stage Disease (PREVEND) prospective cohort, we aimed to investigate the associations of MVX, IVX and MMX with all‐cause mortality, and to determine whether these associations are confounded or modified by thyroid function (FT3, FT4 and TSH) or TPOAb positivity.

## 2 Methods

### 2.1 Study Design and Population

This study followed the STROBE (Strengthening the Reporting of Observational Studies in Epidemiology) guidelines for observational research (Supporting Information S1) [16]. The analysis was based on data from the PREVEND study, a prospective cohort study in the Netherlands designed to investigate the role of urinary albumin excretion in kidney and CVD development. The study's design, recruitment process, and methodology have been described in previous publications [17, 18, 19, 20]. Ethical approval was obtained from the Medical Ethics Committee of the University Medical Center Groningen (Reference: MEC 96/01/022), and all procedures adhered to the principles of the Declaration of Helsinki. Written informed consent was secured from all participants. The study sample consisted of residents of Groningen, Netherlands, selected to reflect the general population. Initially, 8592 individuals participated in the screening phase (1997–1998), with 6894 participants aged 28–75 years included in the baseline assessment between 2001 and 2003. To minimise confounding, individuals using thyroid medications (levothyroxine, triiodothyronine, or anti‐thyroid drugs) or diagnosed with thyroid diseases were excluded. The final analytic cohort comprised 5446 participants with complete data on metabolic malnutrition and inflammation markers (MVX, IVX and MMX), thyroid function measures, relevant confounders, and mortality outcomes, as detailed in Supporting Information S2.

### 2.2 Assessment of Exposures and Other Risk Markers

Data for this study were collected over two outpatient visits, during which information on demographics, lifestyle factors, medical history, physical characteristics, and medication use was recorded. Blood pressure measurements were taken multiple times at each visit, with the final value calculated as the average of the last two readings. Type 2 diabetes (T2D) was diagnosed based on fasting plasma glucose (FPG) levels of ≥ 7.0 mmol/L (126 mg/dL), random plasma glucose levels of ≥ 11.1 mmol/L (200 mg/dL), a self‐reported physician diagnosis, or the initiation of glucose‐lowering medication documented in a central pharmacy database [21]. Body mass index (BMI) was determined by dividing weight (kg) by height squared (m^2^), and alcohol consumption was assessed through self‐reported questionnaires. Venous blood samples were obtained after an overnight fast and processed for biochemical analyses [22, 23, 24, 25, 26]. Serum and EDTA‐anticoagulated plasma were stored at −80°C until further testing. FT3, FT4, TSH and TPOAb were measured using electrochemiluminescent immunoassays on the Roche Modular E170 Analyser (Roche Diagnostics, Mannheim, Germany). The reference ranges used were FT3: 3.1–6.8 pmol/L, FT4: 12–22 pmol/L and TSH: 0.27–4.20 mIU/L, with TPOAb positivity defined as levels ≥ 34 kIU/L.

EDTA‐anticoagulated plasma samples were sent frozen at −80°C to Labcorp (Morrisville, NC, USA) for measurement of metabolic markers using nuclear magnetic resonance (NMR) spectroscopy on a Vantera Clinical NMR Analyser [27]. MVX, IVX and MMX scores were calculated by Labcorp using a proprietary algorithm that was developed using cohorts of subjects at high risk of cardiovascular disease [3]. IVX, the inflammatory component of MVX, is calculated using small HDL particles (S‐HDL‐P; HDL particle size < 9 nm) and GlycA, the latter being an NMR‐based inflammation biomarker whose signals arise from the N‐acetyl methyl group protons of the N‐acetylglucosamine moieties located on the bi‐, tri‐ and tetra‐antennary branches of plasma glycoproteins, mainly α1‐acid glycoprotein, haptoglobin, α1‐antitrypsin, α1‐antichymotrypsin, and transferrin. MMX, the malnutrition component of MVX, is calculated using citrate and the branched chain amino acids, valine, leucine and isoleucine.

The % coefficients of variation (CV) for within‐lab precision with the ranges covering low, medium and high concentration pools for each analyte are as follows: GlycA: 2.0%–2.2%, S‐HDL‐P: 2.5%–4.3%, valine: 2.3%–4.4%, leucine: 5.0%–12.0%, isoleucine: 8.3%–19.7%, citrate: 7.0%–8.4%, MMX: 2.1%–6.1%, IVX: 0.1%–6.5% and MVX: 1.0%–6.2%. IVX and MMX scores are combined to generate the MVX scores. The IVX, MMX and MVX scores are computed using algorithms originally developed by Otvos et al. [3], and modified by Wicks et al. [28] In the present analysis, the algorithm provided by Wicks et al. [28] was used without additional model fitting or coefficient reweighting. The indices were scaled from 1 to 100 with higher values indicating a higher risk of mortality. The algorithms are provided in Supporting Information S3.

Fasting plasma glucose was analysed via dry chemistry methods (Eastman Kodak, Rochester, New York). Serum creatinine was measured using an enzymatic method on a Roche Modular analyser (Roche Diagnostics, Mannheim, Germany). Serum cystatin C was measured using nephelometry (BN II N, Dade Behring Diagnostic, Marburg, Germany), and estimated glomerular filtration rate (GFR) was calculated using the CKD‐EPI combined creatinine‐cystatin C equation [29].

### 2.3 Outcome Ascertainment

Data on all‐cause mortality were obtained from municipality records. We included all deaths that occurred from study enrollment through to 2017.

### 2.4 Statistical Analysis

Variables with skewed distributions, such as triglycerides, GlycA and TSH, were transformed using the natural logarithm to approximate normality. Baseline characteristics were summarised as means with standard deviations (SD) or medians with interquartile ranges (IQR) for continuous variables and as counts with percentages for categorical variables. To explore potential nonlinear dose–response relationships between the exposures and mortality risk, we constructed restricted cubic splines with knots at the 10th, 50th and 90th percentiles of the distributions of the exposures in multivariable adjusted models. Cox proportional hazards models were used to calculate hazard ratios (HRs) with 95% confidence intervals (CIs) for the associations of MVX, IVX and MMX with the risk of all‐cause mortality, after confirmation of no major departure from the proportionality of hazards assumptions [30]. The exposures were modelled as both continuous (per 1 SD increment) and categorical (quartiles defined according to the baseline distribution of their levels) variables. Confounder adjustments were made across five progressively comprehensive models: (Model 1) age and sex; (Model 2) model 1 plus smoking status, history of T2D, systolic blood pressure, total cholesterol, HDL‐C, triglycerides, BMI, estimated GFR, alcohol intake, antihypertensive medication use, and history of CVD; (Model 3) model 2 plus FT3; (Model 4) model 2 plus FT4; and (Model 5) model 2 plus TSH. The selection of confounders was guided by their established roles as risk factors for all‐cause mortality in previous studies including the PREVEND study [20, 31, 32] and observed associations in the dataset [33]. Interaction tests were performed to assess whether the associations of MVX, IVX and MMX with all‐cause mortality were modified by sex, as well as by thyroid function markers (FT3, FT4 and TSH) and TPOAb positivity. FT3, FT4 and TSH were modelled as centered continuous variables to preserve data granularity, minimise information loss associated with categorisation, and improve interpretability. All statistical analyses were conducted using R (version 4.0.4, R Foundation for Statistical Computing, Vienna, Austria) and Stata MP version 18 (StataCorp, College Station, Texas).

## 3 Results

### 3.1 Baseline Characteristics

Baseline descriptive characteristics of the 5446 participants overall and by mortality status at the end of the follow‐up period are presented in Table 1. The mean age of participants at study entry was 54 (SD 12) years and 49.9% were males. Compared to those who were alive at the end of follow‐up period, those who died were older and more likely to be male and current smokers, have higher levels of multimarkers of metabolic malnutrition and inflammation, FT4, BMI, blood pressure and triglycerides, have lower TSH and estimated GFR, and were more likely to have prevalent T2D and CVD and use antihypertensive medication.

**TABLE 1:** Baseline characteristics of participants overall and by status at end of follow‐up.

Variable	Overall (*N* = 5446) Mean (SD) or median (IQR)	Status at end of follow‐up		*p*
		Dead (*N* = 806) Mean (SD) or median (IQR)	Alive (*N* = 4640) Mean (SD) or median (IQR)	
MVX score	44.9 (9.0)	48.8 (9.0)	44.2 (8.9)	< 0.001
IVX score	39.2 (10.5)	43.6 (10.3)	38.4 (10.4)	< 0.001
MMX score	54.3 (6.4)	55.6 (6.7)	54.0 (6.3)	< 0.001
GlycA (μmol/L)	373 [336, 416]	369 [333, 410]	395 [357, 437]	< 0.001
Small HDL particles (μmol/L)	15.92 (2.43)	15.28 (2.44)	16.03 (2.42)	< 0.001
Leucine (μmol/L)	104.42 (27.12)	106.46 (28.32)	104.07 (26.89)	0.02
Valine (μmol/L)	201.60 (38.36)	207.97 (38.66)	200.49 (38.21)	< 0.001
Isoleucine (μmol/L)	58.43 (14.67)	61.99 (15.56)	57.81 (14.42)	< 0.001
Citrate (μmol/L)	2.08 (0.48)	2.28 (0.53)	2.05 (0.46)	< 0.001
FT3 (pmol/L)	4.89 (0.74)	4.85 (1.05)	4.89 (0.67)	0.14
FT4 (pmol/L)	15.59 (2.33)	15.84 (2.50)	15.54 (2.30)	< 0.001
TSH (mU/L)	1.62 [1.11, 2.35]	1.55 [1.02, 2.26]	1.63 [1.13, 2.37]	0.004
TPOAb Positivity, *n* (%)	593 (10.9)	73 (9.1)	520 (11.2)	0.08
**Questionnaire**				
Age (years)	54 (12)	67 (10)	51 (11)	< 0.001
Males, *n* (%)	2717 (49.9)	539 (66.9)	2178 (46.9)	< 0.001
Alcohol consumers, *n* (%)	4086 (75.0)	565 (70.1)	3521 (75.9)	< 0.001
Smokers, *n* (%)				< 0.001
Never smokers	1556 (28.6)	132 (16.4)	1424 (30.7)	
Former smokers	2343 (43.0)	427 (53.0)	1916 (41.3)	
Light current smokers	595 (10.9)	93 (11.5)	502 (10.8)	
Heavy current smokers	952 (17.5)	154 (19.1)	798 (17.2)	
History of T2D, *n* (%)	331 (6.1)	124 (15.4)	207 (4.5)	< 0.001
History of CVD, *n* (%)	155 (2.8)	73 (9.1)	82 (1.8)	< 0.001
Use of antihypertensive medication, *n* (%)	1013 (18.6)	347 (43.1)	666 (14.4)	< 0.001
**Physical measurements**				
BMI (kg/m^2^)	26.7 (4.3)	27.3 (4.3)	26.6 (4.3)	< 0.001
SBP (mmHg)	126 (19)	137 (21)	124 (17)	< 0.001
DBP (mmHg)	73 (9)	76 (9)	73 (9)	< 0.001
**Lipid and renal markers**				
Total cholesterol (mmol/L)	5.43 (1.05)	5.42 (1.10)	5.43 (1.04)	0.73
HDL‐C (mmol/L)	1.26 (0.31)	1.21 (0.33)	1.27 (0.31)	< 0.001
Triglycerides (mmol/L)	1.13 [0.81, 1.62]	1.11 [0.80, 1.60]	1.23 [0.90, 1.72]	< 0.001
Creatinine (mg/dl)	0.83 (0.23)	0.92 (0.45)	0.81 (0.16)	< 0.001
Cystatin C (mg/L)	0.91 (0.21)	1.08 (0.35)	0.88 (0.16)	< 0.001
Estimated GFR (ml/min/1.73 m^2^)	84.1 (10.5)	74.8 (15.8)	85.7 (8.3)	< 0.001

*Note:* Continuous variables are reported as mean (SD) or median (interquartile range) and categorical variables are reported as *n* (%). Former smokers were those who were non‐smokers at the time of study inclusion but had ever smoked in their life; current smokers were those who reported smoking at the time of inclusion; light current smokers were current smokers who reported smoking 10 cigarettes or less per day; and heavy current smokers were current smokers who reported smoking more than 10 cigarettes per day.

Abbreviations: BMI, body mass index; DBP, diastolic blood pressure; FT3, free triiodothyronine; FT4, free thyroxine; GFR, glomerular filtration rate (as calculated using the Chronic Kidney Disease Epidemiology Collaboration combined creatinine–cystatin C equation); HDL‐C, high density lipoprotein cholesterol; IQR, interquartile range; IVX, the inflammation vulnerability index; MMX, the metabolic malnutrition index; MVX, the metabolic vulnerability index; SBP, systolic blood pressure; SD, standard deviation; T2D, type 2 diabetes; TPOAb, thyroid peroxidase antibodies; TSH, thyroid‐stimulating hormone.

### 3.2 Associations of MVX, IVX and MMX With All‐Cause Mortality Risk

**FIGURE 1:**
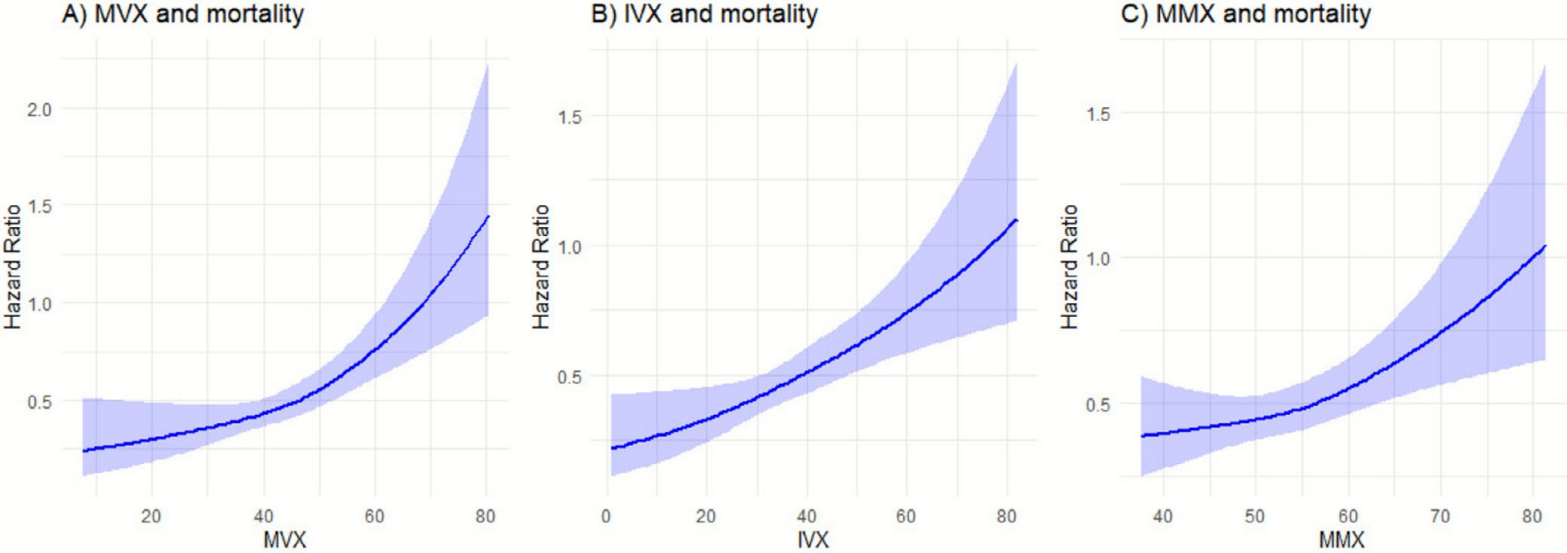
Restricted cubic splines of the hazard ratios of all‐cause mortality with MVX, IVX and MMX. (A) MVX and mortality; (B) IVX and mortality; (C) MMX and mortality. IVX, inflammation vulnerability index; MMX, metabolic malnutrition index; MVX, metabolic vulnerability index. Shaded portions represent the 95% confidence intervals for the spline model (solid line). Models were adjusted for age, sex, smoking status, history of type 2 diabetes, systolic blood pressure, total cholesterol, high‐density lipoprotein cholesterol, triglycerides, body mass index, estimated glomerular filtration rate, alcohol intake, antihypertensive medication use, history of cardiovascular disease and TPOAb positivity.

**FIGURE 2:**
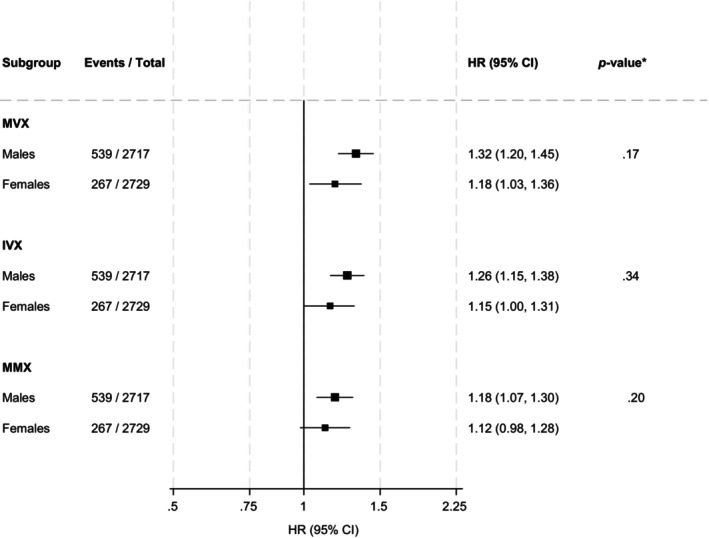
Associations of MVX, IVX and MMX with mortality by sex. HRs are per 1 standard deviation increase in each exposure. CI, confidence interval; HR, hazard ratio; IVX, inflammation vulnerability index; MMX, metabolic malnutrition index; MVX, metabolic vulnerability index. Models were adjusted for age, smoking status, history of type 2 diabetes, systolic blood pressure, total cholesterol, high‐density lipoprotein cholesterol, triglycerides, body mass index, estimated glomerular filtration rate, alcohol intake, antihypertensive medication use, history of cardiovascular disease and TPOAb positivity. *p‐values for interaction.

During a median follow‐up of 14.1 (IQR, 11.9–14.7) years, corresponding to 68862.8 person‐years at risk, 806 deaths (annual rate 11.7/1000 person‐years at risk; 95% CI: 10.9–12.5) were recorded. In dose–response spline analyses, increasing levels of MVX, IVX and MMX were each related to an increasing risk of mortality in a graded fashion (Figure 1). Table 2 shows the associations of MVX, IVX and MMX with the risk of mortality. In analysis adjusted for model 2 covariates (age, sex, smoking status, history of T2D, SBP, total cholesterol, HDL‐C, triglycerides, BMI, estimated GFR, alcohol intake, antihypertensive medication use, history of CVD, and TPOAb positivity), the HR (95% CI) of mortality was 1.28 (1.18–1.38) per 1 SD increase in MVX. The corresponding HRs (95% CIs) were 1.23 (1.14–1.32) and 1.16 (1.07–1.25) for IVX and MMX, respectively. The HRs were materially unchanged for all exposures on further adjustment for FT3, FT4 and TSH: 1.28 (1.18–1.39), 1.23 (1.14–1.32) and 1.16 (1.07–1.25), respectively. Comparing quartile 4 with quartile 1, the HRs (95% CIs) of mortality for MVX, IVX and MMX were 1.88 (1.48–2.39), 1.72 (1.36–2.18) and 1.42 (1.14–1.78), respectively in analysis adjusted for model 2 covariates. The HRs remained unchanged on further adjustment for FT3, FT4 and TSH. There was no significant evidence that sex modified the associations of MVX, IVX and MMX with mortality risk (Figure 2).

**TABLE 2:** Associations of MVX, IVX and MMX with all‐cause mortality.

Exposures	Events/Total	Model 1		Model 2		Model 3		Model 4		Model 5	
		HR (95% CI)	*p*	HR (95% CI)	*p*	HR (95% CI)	*p*	HR (95% CI)	*p*	HR (95% CI)	*p*
**MVX**											
Per 1 SD increase	806/5446	1.39 (1.29–1.50)	< 0.001	1.28 (1.18–1.38)	< 0.001	1.28 (1.18–1.39)	< 0.001	1.27 (1.18–1.38)	< 0.001	1.28 (1.18–1.39)	< 0.001
Q1 (< 39)	106/1377	ref		ref		ref		ref		ref	
Q2 (39–45)	153/1349	1.26 (0.98–1.62)	0.07	1.19 (0.93–1.53)	0.18	1.19 (0.93–1.53)	0.18	1.19 (0.92–1.52)	0.18	1.19 (0.92–1.53)	0.18
Q3 (45.1–50.9)	210/1365	1.49 (1.17–1.89)	< 0.001	1.30 (1.02–1.65)	0.04	1.30 (1.02–1.66)	0.03	1.29 (1.01–1.65)	0.04	1.30 (1.02–1.65)	0.04
Q4 (≥ 51)	337/1355	2.32 (1.84–2.92)	< 0.001	1.88 (1.48–2.39)	< 0.001	1.89 (1.48–2.39)	< 0.001	1.87 (1.47–2.38)	< 0.001	1.88 (1.48–2.39)	< 0.001
**IVX**											
Per 1 SD increase	806/5446	1.34 (1.25–1.44)	< 0.001	1.23 (1.14–1.32)	< 0.001	1.23 (1.14–1.33)	< 0.001	1.22 (1.13–1.32)	< 0.001	1.23 (1.14–1.32)	< 0.001
Q1 (< 32.2)	104/1366	ref		ref		ref		ref		ref	
Q2 (32.2–39.2)	160/1375	1.20 (0.93–1.53)	0.16	1.12 (0.87–1.44)	0.37	1.12 (0.87–1.45)	0.37	1.13 (0.88–1.45)	0.35	1.12 (0.87–1.44)	0.37
Q3 (39.3–46)	209/1350	1.52 (1.20–1.93)	< 0.001	1.32 (1.04–1.68)	0.03	1.32 (1.04–1.69)	0.03	1.32 (1.03–1.68)	0.03	1.32 (1.04–1.68)	0.03
Q4 (≥ 46.1)	333/1355	2.16 (1.72–2.71)	< 0.001	1.72 (1.36–2.18)	< 0.001	1.73 (1.36–2.19)	< 0.001	1.72 (1.36–2.18)	< 0.001	1.72 (1.36–2.19)	< 0.001
**MMX**											
Per 1 SD increase	806/5446	1.17 (1.09–1.26)	< 0.001	1.16 (1.07–1.25)	< 0.001	1.16 (1.07–1.25)	< 0.001	1.16 (1.07–1.25)	< 0.001	1.16 (1.07–1.25)	< 0.001
Q1 (< 49.9)	156/1392	ref		ref		ref		ref		ref	
Q2 (49.9–53.7)	180/1356	1.05 (0.85–1.31)	0.65	1.07 (0.86–1.32)	0.57	1.07 (0.86–1.32)	0.57	1.07 (0.86–1.33)	0.56	1.07 (0.86–1.33)	0.56
Q3 (53.8–58.1)	210/1342	1.21 (0.98–1.49)	0.08	1.18 (0.96–1.47)	0.12	1.18 (0.96–1.47)	0.12	1.17 (0.95–1.45)	0.14	1.18 (0.96–1.47)	0.12
Q4 (≥ 58.2)	260/1356	1.47 (1.19–1.81)	< 0.001	1.42 (1.14–1.78)	0.002	1.42 (1.14–1.78)	0.002	1.42 (1.14–1.77)	0.002	1.43 (1.14–1.78)	0.002

*Note:* Model 1: Age and sex. Model 2: Model 1 plus smoking status, history of type 2 diabetes, systolic blood pressure, total cholesterol, high‐density lipoprotein cholesterol, triglycerides, BMI, estimated GFR, alcohol intake, antihypertensive medication use, history of cardiovascular disease and TPOAb positivity. Model 3: Model 2 plus FT3. Model 4: Model 2 plus FT4. Model 5: Model 2 plus TSH. Abbreviations: CI, confidence interval; HR, hazard ratio; IVX, inflammation vulnerability index; MMX, metabolic malnutrition index; MVX, metabolic vulnerability index; Q, quartile; SD, standard deviation.

There was no significant evidence that FT3, FT4 or TSH, when modelled as centered continuous variables, modified the associations of MVX, IVX or MMX with the risk of all‐cause mortality (Table 3). Similarly, no effect modification was observed by TPOAb positivity (Supporting Information S4).

**TABLE 3:** Associations of MVX, IVX and MMX with all‐cause mortality according to centred continuous FT3, FT4 and TSH.

Exposure	HR (95% CI)	*p*	*p* for interaction
**Centered FT3**			
MVX	1.29 (1.18–1.39)	< 0.001	0.46
IVX	1.24 (1.15–1.34)	< 0.001	0.17
MMX	1.16 (1.07–1.25)	< 0.001	0.54
**Centered FT4**			
MVX	1.27 (1.17–1.38)	< 0.001	0.34
IVX	1.22 (1.13–1.32)	< 0.001	0.60
MMX	1.15 (1.07–1.25)	< 0.001	0.19
**Centered TSH**			
MVX	1.28 (1.18–1.38)	< 0.001	0.16
IVX	1.23 (1.14–1.32)	< 0.001	0.20
MMX	1.16 (1.08–1.26)	< 0.001	0.23

*Note:* FT3, FT4 and TSH are centered by subtracting their respective means from each observed value. Hazard ratios are per 1 standard deviation increase in each exposure. Analyses were adjusted for age, sex, smoking status, history of type 2 diabetes, systolic blood pressure, total cholesterol, high‐density lipoprotein cholesterol, triglycerides, body mass index, estimated glomerular filtration rate, alcohol intake, antihypertensive medication use, history of cardiovascular disease and TPOAb positivity.

Abbreviations: CI, confidence interval; FT4, free thyroxine; FT3, free triiodothyronine; HR, hazard ratio; IVX, inflammation vulnerability index; MMX, metabolic malnutrition index; MVX, metabolic vulnerability index; TSH, thyroid‐stimulating hormone.

### 3.3 Associations of MVX, IVX and MMX With Cause‐Specific Mortality Risk

During the follow‐up period, 192 deaths were attributed to cardiovascular causes and 614 to non‐cardiovascular causes. MVX, IVX and MMX were all significantly associated with increased risk of CVD and non‐CVD mortality. The strength and direction of these associations were broadly consistent with those observed for all‐cause mortality. Notably, the association between MMX and CVD mortality was more modest (Supporting Information S5 and S6).

## 4 Discussion

In this prospective cohort study, we examined the associations of the MVX and its subcomponents—the IVX and MMX—with all‐cause mortality risk, while assessing the potential role of thyroid function as a confounder and effect modifier. Our findings show that participants who died during follow‐up had significantly higher baseline levels of MVX, IVX, MMX and FT4 compared to those who survived. MVX, IVX and MMX were each positively associated with all‐cause mortality, consistent with graded dose–response relationships, with MVX exhibiting the strongest association, followed by IVX and MMX. These associations remained persistent after adjusting for markers of thyroid function (TSH, FT4 and FT3). Furthermore, thyroid function as well as TPOAb positivity did not modify the associations between MVX, IVX and MMX with mortality. The associations were also not modified by sex. In additional analyses, MVX, IVX and MMX were each significantly associated with both cardiovascular and non‐cardiovascular mortality, reinforcing their robustness as risk markers across cause‐specific mortality outcomes.

Our study builds upon seminal work by Otvos and colleagues [3], who first derived MVX from six simultaneously measured serum biomarkers linked to metabolic malnutrition and inflammation syndromes. Their study, conducted in two longitudinal cohorts of cardiac catheterisation patients, demonstrated strong, graded associations between MVX, IVX and MMX multimarker scores and all‐cause mortality. Notably, these associations remained consistent across different age groups, sexes and comorbidities. Furthermore, our findings align with recent work by Conners and colleagues [4], who examined the prognostic value of MVX in a cohort of patients with heart failure. Their study demonstrated that MVX was strongly associated with mortality, independent of established clinical risk factors, and improved mortality risk classification beyond clinically validated markers. Our study confirms and extends these findings by demonstrating for the first time similar associations in a general population‐based cohort. Collectively, these findings reinforce the prognostic utility of MVX and its subcomponents in diverse populations and disease settings, supporting its potential role as a robust biomarker for metabolic vulnerability and mortality risk. A previous study amongst PREVEND participants found that higher FT4 levels were associated with all‐cause mortality, while FT3 was linked to mortality risk in women only [34]. More recently, an individual participant meta‐analysis identified the lowest risk for all‐cause mortality at an FT4 level around the 20th to 40th percentile and a TSH level around the 60th to 80th percentile [7]. Importantly, our findings demonstrate that the associations of MVX, IVX and MMX with all‐cause mortality are independent of and are not modified by thyroid function parameters (FT3, FT4 and TSH).

These findings align with prior research suggesting that systemic metabolic dysfunction, driven by inflammation and protein‐energy wasting, is a key determinant of survival outcomes [35]. One plausible explanation for our results is that MVX, IVX and MMX capture underlying metabolic and inflammatory derangements linked to chronic disease progression [3, 36]. The biomarkers comprising these indices—including inflammatory markers (e.g., GlycA) and metabolic indicators (e.g., branched‐chain amino acids and citrate) – reflect systemic inflammation, oxidative stress, and impaired metabolic resilience [3], all of which are established contributors to increased mortality risk [32, 37, 38]. Chronic inflammation and protein‐energy malnutrition accelerate muscle catabolism, endothelial dysfunction, and immune dysregulation, predisposing individuals to both cardiovascular and non‐cardiovascular causes of death. Although thyroid hormones play a central role in metabolic regulation [5], we found no consistent evidence that FT3, FT4 or TSH modified the associations of MVX, IVX or MMX with mortality. This may suggest that the metabolic vulnerability captured by these indices operates independently of thyroid status, or that any contribution of thyroid dysfunction is already encompassed within the broader metabolic and inflammatory pathways reflected by MVX. However, emerging evidence supports a potential interplay between thyroid function and metabolic vulnerability. A recent cross‐sectional analysis from the Brazilian Longitudinal Study of Adult Health (ELSA‐Brasil) found that lower FT3 levels and a reduced FT3:FT4 ratio were associated with higher MVX scores—even amongst individuals with normal thyroid function and those with cardiometabolic diseases [39]. These results align with the biological plausibility that alterations in thyroid hormone activity may influence systemic metabolic balance, which is captured by composite indices like MVX.

Despite this cross‐sectional evidence, our prospective findings suggest that thyroid function does not substantially confound or modify the relationship between metabolic vulnerability and mortality risk in a general population cohort. This core negative finding highlights the robustness of MVX and its subcomponents as risk markers of adverse outcomes, independent of thyroid status. One potential explanation for this lack of interaction is the low prevalence of NTIS in the general population. NTIS is typically seen in acutely or chronically ill hospitalised patients and is uncommon in community‐based cohorts [8, 40]. As such, the absence of widespread NTIS in our study population may have limited the opportunity to detect thyroid function–related effect modification. Moreover, several components of MVX, including GlycA and inflammatory glycoproteins, reflect biological pathways that overlap with those regulated by thyroid hormones [41]. For instance, thyroid dysfunction, particularly low FT3 states, is associated with systemic inflammation, dyslipidemia, and insulin resistance [42, 43], which are already encompassed by MVX. It is therefore plausible that MVX integrates much of the metabolic and inflammatory perturbations induced by thyroid dysfunction, rendering thyroid hormones redundant as additional modifiers of mortality risk. Nonetheless, it remains plausible that subclinical or longitudinal changes in thyroid function may influence the evolution of metabolic vulnerability over time. Further mechanistic and prospective studies are needed to clarify these complex interrelations and determine whether subtle thyroid dysfunction may act upstream in the causal pathway linking metabolic dysregulation and adverse outcomes.

Our findings highlight the critical role of systemic metabolic vulnerability, as captured by the MVX, IVX and MMX multimarkers, as a risk indicator for mortality. The strong and consistent associations observed, independent of thyroid function, suggest that metabolic malnutrition and inflammation are important contributors to mortality risk in the general population. Although we did not formally assess the incremental value of these markers for clinical risk prediction, the observed graded dose–response relationships indicate their potential utility as indicators of underlying health risk. These results support further investigation into the prognostic value of MVX and its components, including whether they could enhance existing risk models or serve as biomarkers for monitoring metabolic health over time in general population participants. Future research should also examine whether targeting metabolic vulnerability through therapeutic interventions may reduce mortality risk. Although MVX, IVX and MMX show strong and independent associations with mortality risk, their integration into routine clinical practice is currently constrained by practical limitations. These multimarker indices require NMR spectroscopy and proprietary algorithms, which are available in major clinical laboratories in North America but have more limited accessibility elsewhere. The cost and infrastructure requirements of NMR‐based assays may pose additional barriers to widespread clinical adoption. As NMR technology becomes more widely implemented, particularly in European clinical laboratories, access to such algorithms is likely to expand. Future research should focus on developing simplified or alternative versions of these indices using more commonly available biomarkers, and on evaluating the cost‐effectiveness and clinical utility of MVX‐based approaches in high‐risk populations where metabolic vulnerability may be especially relevant.

### 4.1 Strengths and Limitations

This study represents the first prospective evaluation of the associations between MVX and its subcomponents (IVX and MMX) with mortality in a general population, while also examining the potential role of thyroid function. Key strengths include the large cohort size, extended follow‐up duration, and comprehensive adjustment for potential confounders. The study also benefits from robust statistical analyses, including dose–response evaluations and assessment of interactions, which enhance the reliability of the findings. However, several limitations should be acknowledged. As noted in previous research [3], the equations used to derive MVX, IVX and MMX are still evolving, with potential limitations in the accuracy of certain biomarkers, such as branched‐chain amino acids, and the possible omission of additional relevant markers. Like all observational studies, this investigation is susceptible to measurement errors, unmeasured confounding, and the inability to establish causality. We did not adjust for high‐sensitivity C‐reactive protein (hsCRP) in our multivariable models because GlycA, an integral component of the MVX algorithm, is itself a robust and validated biomarker of systemic inflammation. GlycA has been shown to capture both acute and chronic inflammatory states and is strongly associated with adverse cardiometabolic outcomes [32, 38]. Moreover, in our dataset, GlycA demonstrated a strong positive correlation with hsCRP (Pearson's *r* = 0.66). Given this substantial overlap in the inflammatory signal captured by both biomarkers, additional adjustment for hsCRP may have introduced issues of over‐adjustment, which could obscure the true associations between MVX and mortality risk. Moreover, the association between GlycA and all‐cause mortality is at least as strong, if not stronger, than that of hsCRP [32]. Additionally, biomarker levels were measured only at baseline, limiting insights into how changes over time might influence mortality risk. Finally, given that the study population was predominantly of White ethnicity, the generalisability of the findings to other racial and ethnic groups remains uncertain.

## 5 Conclusion

This study provides strong evidence that MVX and its subcomponents (IVX and MMX) are independently associated with all‐cause mortality, consistent with graded dose–response relationships. These associations were not confounded or modified by thyroid function markers. Moreover, MVX, IVX and MMX were also significantly associated with both cardiovascular and non‐cardiovascular mortality, underscoring their robustness as risk markers across different causes of death. These findings highlight metabolic vulnerability as a key determinant of survival. Further research is needed to explore the clinical applications of MVX and whether interventions targeting metabolic vulnerability can improve survival outcomes.

## Author Contributions

Setor K. Kunutsor (conceptualisation [lead], data curation [lead], formal analysis [lead], investigation [lead], methodology [lead], project administration [equal], software [lead], supervision [equal], validation [lead], visualisation [lead], writing – original draft [lead], writing – review and editing [lead]), Reyhaneh Rikhtehgaran (data curation [supporting], formal analysis [equal], investigation [supporting], methodology [supporting], software [equal], validation [equal], visualisation [equal], writing – review and editing [supporting]), Yanning Xu (data curation [supporting], investigation [supporting], methodology [supporting], visualisation [supporting], writing – review and editing [supporting]), Margery A. Connelly (conceptualisation [supporting], data curation [equal], funding acquisition [supporting], investigation [supporting], methodology [supporting], project administration [supporting], resources [supporting], validation [supporting], writing – review and editing [supporting]), Irina Shalaurova (conceptualisation [supporting], data curation [equal], investigation [supporting], methodology [supporting], resources [supporting], writing – review and editing [supporting]), Layal Chaker (conceptualisation [supporting], investigation [supporting], methodology [supporting], visualisation [supporting], writing – review and editing [supporting]), Stephan J. L. Bakker (conceptualisation [equal], data curation [equal], investigation [supporting], methodology [supporting], resources [equal], validation [equal], writing – review and editing [supporting]), Robin P. F. Dullaart (conceptualisation [equal], investigation [equal], methodology [equal], software [lead], project administration [lead], supervision [equal], validation [equal], writing – review and editing [equal]).

## Funding

The Dutch Kidney Foundation supported the infrastructure of the PREVEND program from 1997 to 2003 (Grant E.033). The University Medical Center Groningen supported the infrastructure from 2003 to 2006. Dade Behring, Ausam, Roche, and Abbott financed laboratory equipment and reagents by which various laboratory determinations could be performed. The Dutch Heart Foundation supported studies on lipid metabolism (Grant 2001‐005). The contents of this paper are solely the responsibility of the authors and do not represent the views of the Sponsors. The sponsors did not participate in the design and conduct of the study; collection, management, analysis and interpretation of the data; or preparation of the manuscript.

## Conflicts of Interest

Margery A. Connelly is an employee of and holds stock in Labcorp. Irina Shalaurova is an employee of Labcorp. All other authors declare no conflicts of interest.

## Supporting information


**Supporting Information: S1** Derivation of the analytic sample.
**Supporting Information: S2** STROBE 2007 Statement—Checklist of items that should be included in reports of cohort studies.
**Supporting Information: S3** Formulas for computing MVX, IVX and MMX.
**Supporting Information: S4** Associations of MVX, IVX and MMX with mortality by TPOAb positivity.
**Supporting Information: S5** Associations of MVX, IVX and MMX with CVD mortality.
**Supporting Information: S6** Associations of MVX, IVX and MMX with non‐CVD mortality.

## Data Availability

All data generated or analysed during this study are included in this published article or are available from the corresponding author upon reasonable request.
