# Correction to ʻAssociations of Multimarkers of Metabolic Malnutrition and Inflammation With All‐Cause Mortality and Their Interplay With Thyroid Functionʼ

**DOI:** 10.1002/edm2.70200

**Published:** 2026-03-06

S. K. Kunutsor, R. Rikhtehgaran, Y. Xu, et al., “Associations of Multimarkers of Metabolic Malnutrition and Inflammation With All‐Cause Mortality and Their Interplay With Thyroid Function,” Endocrinology, Diabetes & Metabolism 9, no. 2 (2026): e70162, 10.1002/edm2.70162.

In Table 1 of the above article, the values for GlycA and triglycerides were incorrectly swapped between the alive and dead groups. This error is confined to Table 1. The corrected Table 1 is provided below and does not affect the analyses, results, interpretation, or conclusions of the article.

**TABLE 1:** Baseline characteristics of participants overall and by status at end of follow‐up.

Variable	Overall (*N* = 5446) mean (SD) or median (IQR)	Status at end of follow‐up		*p*
		Dead (*N* = 806) mean (SD) or median (IQR)	Alive (*N* = 4640) mean (SD) or median (IQR)	
MVX score	44.9 (9.0)	48.8 (9.0)	44.2 (8.9)	< 0.001
IVX score	39.2 (10.5)	43.6 (10.3)	38.4 (10.4)	< 0.001
MMX score	54.3 (6.4)	55.6 (6.7)	54.0 (6.3)	< 0.001
GlycA (μmol/L)	373 (336, 416)	395 (357, 437)	369 (333, 410)	< 0.001
Small HDL particles (μmol/L)	15.92 (2.43)	15.28 (2.44)	16.03 (2.42)	< 0.001
Leucine (μmol/L)	104.42 (27.12)	106.46 (28.32)	104.07 (26.89)	0.02
Valine (μmol/L)	201.60 (38.36)	207.97 (38.66)	200.49 (38.21)	< 0.001
Isoleucine (μmol/L)	58.43 (14.67)	61.99 (15.56)	57.81 (14.42)	< 0.001
Citrate (μmol/L)	2.08 (0.48)	2.28 (0.53)	2.05 (0.46)	< 0.001
FT3 (pmol/L)	4.89 (0.74)	4.85 (1.05)	4.89 (0.67)	0.14
FT4 (pmol/L)	15.59 (2.33)	15.84 (2.50)	15.54 (2.30)	< 0.001
TSH (mU/L)	1.62 (1.11, 2.35)	1.55 (1.02, 2.26)	1.63 (1.13, 2.37)	0.004
TPOAb positivity, *n* (%)	593 (10.9)	73 (9.1)	520 (11.2)	0.08
*Questionnaire*				
Age (years)	54 (12)	67 (10)	51 (11)	< 0.001
Males, *n* (%)	2717 (49.9)	539 (66.9)	2178 (46.9)	< 0.001
Alcohol consumers, *n* (%)	4086 (75.0)	565 (70.1)	3521 (75.9)	< 0.001
Smokers, *n* (%)				< 0.001
Never smokers	1556 (28.6)	132 (16.4)	1424 (30.7)	
Former smokers	2343 (43.0)	427 (53.0)	1916 (41.3)	
Light current smokers	595 (10.9)	93 (11.5)	502 (10.8)	
Heavy current smokers	952 (17.5)	154 (19.1)	798 (17.2)	
History of T2D, *n* (%)	331 (6.1)	124 (15.4)	207 (4.5)	< 0.001
History of CVD, *n* (%)	155 (2.8)	73 (9.1)	82 (1.8)	< 0.001
Use of antihypertensive medication, *n* (%)	1013 (18.6)	347 (43.1)	666 (14.4)	< 0.001
*Physical measurements*				
BMI (kg/m^2^)	26.7 (4.3)	27.3 (4.3)	26.6 (4.3)	< 0.001
SBP (mmHg)	126 (19)	137 (21)	124 (17)	< 0.001
DBP (mmHg)	73 (9)	76 (9)	73 (9)	< 0.001
*Lipid and renal markers*				
Total cholesterol (mmol/L)	5.43 (1.05)	5.42 (1.10)	5.43 (1.04)	0.73
HDL‐C (mmol/L)	1.26 (0.31)	1.21 (0.33)	1.27 (0.31)	< 0.001
Triglycerides (mmol/L)	1.13 (0.81, 1.62)	1.23 (0.90, 1.72)	1.11 (0.80, 1.60)	< 0.001
Creatinine (mg/dl)	0.83 (0.23)	0.92 (0.45)	0.81 (0.16)	< 0.001
Cystatin C (mg/L)	0.91 (0.21)	1.08 (0.35)	0.88 (0.16)	< 0.001
Estimated GFR (ml/min/1.73m^2^)	84.1 (10.5)	74.8 (15.8)	85.7 (8.3)	< 0.001

*Note:* Continuous variables are reported as mean (SD) or median (interquartile range) and categorical variables are reported as *n* (%). Former smokers were those who were non‐smokers at the time of study inclusion but had ever smoked in their life; current smokers were those who reported smoking at the time of inclusion; light current smokers were current smokers who reported smoking 10 cigarettes or less per day; and heavy current smokers were current smokers who reported smoking more than 10 cigarettes per day.

Abbreviations: BMI, body mass index; DBP, diastolic blood pressure; FT3, free triiodothyronine; FT4, free thyroxine; GFR, glomerular filtration rate (as calculated using the chronic kidney disease epidemiology collaboration combined creatinine–cystatin C equation); HDL‐C, high density lipoprotein cholesterol; IQR, interquartile range; IVX, the inflammation vulnerability index; MMX, the metabolic malnutrition index; MVX, the metabolic vulnerability index; SBP, systolic blood pressure; SD, standard deviation; T2D, type 2 diabetes; TPOAb, thyroid peroxidase antibodies; TSH, thyroid‐stimulating hormone.

We apologize for this error.

