# Supplementary material for: Associations of Multimarkers of Metabolic Malnutrition and Inflammation With All‐Cause Mortality and Their Interplay With Thyroid Function
Source: Endocrinol Diabetes Metab. 2026 Feb 6;9(2):e70162. doi: 10.1002/edm2.70162 (PMC12880609; doi:10.1002/edm2.70162)
Supplement: Supplementary file 1 — Supporting Information: S1 Derivation of the analytic sample. Supporting Information: S2 STROBE 2007 Statement—Checklist of items that should be included in reports of cohort studies. Supporting Information: S3 Formulas for computing MVX, IVX and MMX. Supporting Information: S4 Associations of MVX, IVX and MMX with mortality by TPOAb positivity. Supporting Information: S5 Associations of MVX, IVX and MMX with CVD mortality. Supporting Information: S6 Associations of MVX, IVX and MMX with non‐CVD mortality. [file EDM2-9-e70162-s001.docx]

**Supplementary materials**

| **Supplementary Material 1** | Derivation of the analytic sample |
| --- | --- |
| **Supplementary Material 2** | STROBE 2007 Statement—Checklist of items that should be included in reports of cohort studies |
| **Supplementary Material 3** | Formulas for computing MVX, IVX, and MMX |
| **Supplementary Material 4** | Associations of MVX, IVX, and MMX with mortality by TPOAb positivity |
| **Supplementary Material 5** | Associations of MVX, IVX and MMX with CVD mortality |
| **Supplementary Material 6** | Associations of MVX, IVX and MMX with non-CVD mortality |

**Supplemental Material 1.** Derivation of the analytic sample

**N=6,894**
Eligible PREVEND participants

**N=127**
individuals with thyroid disease or on thyroid medications

**N=6,767**
Participants free of thyroid disease

**N=1,321**
No information on exposures, thyroid function, covariates and outcome

**N=5,446**
Participants included in the analysis of MVX, IVX, and MMX; thyroid function and all-cause mortality

IVX, inflammation vulnerability index; MMX, metabolic malnutrition index; MVX, metabolic vulnerability index

**Supplemental Material 2.** STROBE 2007 Statement—Checklist of items that should be included in reports of cohort studies

| **Section/Topic** | **Item #** | **Recommendation** | **Reported on page #** |
| --- | --- | --- | --- |
| **Title and abstract** | 1 | (*a*) Indicate the study’s design with a commonly used term in the title or the abstract | Page 1 |
|  |  | (*b*) Provide in the abstract an informative and balanced summary of what was done and what was found | Page 2 |
| **Introduction** | | |  |
| Background/rationale | 2 | Explain the scientific background and rationale for the investigation being reported | Page 4 |
| Objectives | 3 | State specific objectives, including any prespecified hypotheses | Page 4 |
| **Methods** | | |  |
| Study design | 4 | Present key elements of study design early in the paper | Study design and population |
| Setting | 5 | Describe the setting, locations, and relevant dates, including periods of recruitment, exposure, follow-up, and data collection | Study design and population |
| Participants | 6 | (*a*) Give the eligibility criteria, and the sources and methods of selection of participants. Describe methods of follow-up | Study design and population |
|  |  | (*b*) For matched studies, give matching criteria and number of exposed and unexposed | Not applicable |
| Variables | 7 | Clearly define all outcomes, exposures, predictors, potential confounders, and effect modifiers. Give diagnostic criteria, if applicable | Assessment of exposures and other risk markers |
| Data sources/ measurement | 8* | For each variable of interest, give sources of data and details of methods of assessment (measurement). Describe comparability of assessment methods if there is more than one group | Assessment of exposures and other risk markers |
| Bias | 9 | Describe any efforts to address potential sources of bias | Statistical analyses |
| Study size | 10 | Explain how the study size was arrived at | Statistical analyses |
| Quantitative variables | 11 | Explain how quantitative variables were handled in the analyses. If applicable, describe which groupings were chosen and why | Statistical analyses |
| Statistical methods | 12 | (*a*) Describe all statistical methods, including those used to control for confounding | Statistical analyses |
|  |  | (*b*) Describe any methods used to examine subgroups and interactions | Statistical analyses |
|  |  | (*c*) Explain how missing data were addressed | Not applicable |
|  |  | (*d*) If applicable, explain how loss to follow-up was addressed | Not applicable |
|  |  | (*e*) Describe any sensitivity analyses | Statistical analyses |
| **Results** | | |  |
| Participants | 13* | (a) Report numbers of individuals at each stage of study—eg numbers potentially eligible, examined for eligibility, confirmed eligible, included in the study, completing follow-up, and analysed | Study design and population |
|  |  | (b) Give reasons for non-participation at each stage | Study design and population |
|  |  | (c) Consider use of a flow diagram | Study design and population |
| Descriptive data | 14* | (a) Give characteristics of study participants (eg demographic, clinical, social) and information on exposures and potential confounders | Results; Table 1 |
|  |  | (b) Indicate number of participants with missing data for each variable of interest |  |
|  |  | (c) Summarise follow-up time (eg, average and total amount) | Results |
| Outcome data | 15* | Report numbers of outcome events or summary measures over time | Results |
| Main results | 16 | (*a*) Give unadjusted estimates and, if applicable, confounder-adjusted estimates and their precision (eg, 95% confidence interval). Make clear which confounders were adjusted for and why they were included | Results; Figure 1; Tables 2-3; Supplementary Materials 3-6 |
|  |  | (*b*) Report category boundaries when continuous variables were categorized | Results; Tables 2-3. Figures 1-2  Supplementary Materials 3-6 |
|  |  | (*c*) If relevant, consider translating estimates of relative risk into absolute risk for a meaningful time period |  |
| Other analyses | 17 | Report other analyses done—eg analyses of subgroups and interactions, and sensitivity analyses | Results |
| **Discussion** |  |  |  |
| Key results | 18 | Summarise key results with reference to study objectives | Discussion |
| **Limitations** |  |  |  |
| Interpretation | 20 | Give a cautious overall interpretation of results considering objectives, limitations, multiplicity of analyses, results from similar studies, and other relevant evidence | Discussion |
| Generalisability | 21 | Discuss the generalisability (external validity) of the study results | Discussion |
| **Other information** |  |  |  |
| Funding | 22 | Give the source of funding and the role of the funders for the present study and, if applicable, for the original study on which the present article is based | Title page |

**Supplemental Material 3.** Formulas for computing MVX, IVX, and MMX

IVX = 9 − 0.0027 *GlycA* − 0.46079 *sHDLP* + 0.0006325 *GlycA* × *sHDLP*

IVX_min_ = 2.0 ⇒ score = 1

IVX_max_ = 8.3 ⇒ score = 100

MMX = (0.75097 [4 − 0.02234 *Leu* + 0.0000528 *Leu^2^*]) + (0.55737 [7 − 0.02895 *Val* + 0.0000608 *Val^2^*]) + (0.00867 *Ile*) + (0.65649 [1 + 0.0025 *Cit* + 0.0000167 *Cit^2^*])

MMX_min_ = 1.281 ⇒ score = 1

MMX_max_ = 2.0 ⇒ score = 100

MVX = 2.72923 IVX + 11.96062 lnMMX − 1.12749 IVX × lnMMX

MVX_min_ = 20.3 ⇒ score = 1

MVX_max_ = 28.0 ⇒ score = 100

Cit, citrate; Ile, isoleucine; Leu, leucine; sHDLP, small high-density lipoprotein particle; Val, valine

The subscripts min and max refer to the minimum and maximum values of the corresponding index.

This algorithm was developed by Wicks TR, Shalaurova I, Wolska A, et al. Endogenous Ketone Bodies Are Associated with Metabolic Vulnerability and Disability in Multiple Sclerosis. *Nutrients*. Feb 11 2025;17(4)

**Supplemental Material 4.** Associations of MVX, IVX, and MMX with mortality by TPOAb positivity

HRs are per 1 standard deviation increase in each exposure

CI, confidence interval; HR, hazard ratio; IVX, inflammation vulnerability index; MMX, metabolic malnutrition index; MVX, metabolic vulnerability index; TPOAb, thyroid peroxidase antibodies

Models were adjusted for age, sex, smoking status, history of type 2 diabetes, systolic blood pressure, total cholesterol, high-density lipoprotein cholesterol, triglycerides, body mass index, estimated glomerular filtration rate, alcohol intake, antihypertensive medication use, and history of cardiovascular disease

*, *p*-values for interaction

**Supplemental Material 5.** Associations of MVX, IVX and MMX with CVD mortality

|  |  | **Model 1** |  | **Model 2** |  | **Model 3** |  | **Model 4** |  | **Model 5** |  |
| --- | --- | --- | --- | --- | --- | --- | --- | --- | --- | --- | --- |
| **Exposures** | **Events/**  **Total** | **HR (95% CI)** | ***p-*value** | **HR (95% CI)** | ***p-*value** | **HR (95% CI)** | ***p-*value** | **HR (95% CI)** | ***p-*value** | **HR (95% CI)** | ***p-*value** |
| **MVX** |  |  |  |  |  |  |  |  |  |  |  |
| Per 1 SD increase | 192 / 5437 | 1.48 (1.27 – 1.72) | < .001 | 1.29 (1.09 – 1.51) | .002 | 1.28 (1.09 – 1.51) | .003 | 1.28 (1.08 – 1.50) | .003 | 1.29 (1.09 – 1.51) | .002 |
| Q1 (< 39) | 19 / 1373 | ref |  | ref |  | ref |  | ref |  | ref |  |
| Q2 (39-45) | 31 / 1348 | 1.24 (0.70 – 2.20) | .46 | 1.17 (0.66 – 2.09) | .59 | 1.17 (0.65 – 2.08) | .60 | 1.16 (0.65 – 2.06) | .62 | 1.17 (0.66 – 2.08) | .60 |
| Q3 (45.1-50.9) | 54 / 1362 | 1.74 (1.02 – 2.97) | .041 | 1.54 (0.89 – 2.65) | .12 | 1.53 (0.89 – 2.63) | .13 | 1.51 (0.88 – 2.60) | .14 | 1.55 (0.90 – 2.66) | .11 |
| Q4 (≥ 51) | 88 / 1354 | 2.59 (1.54 – 4.35) | < .001 | 1.90 (1.11 – 3.23) | .019 | 1.88 (1.10 – 3.20) | .021 | 1.86 (1.09 – 3.17) | .023 | 1.90 (1.12 – 3.24) | .018 |
| **IVX** |  |  |  |  |  |  |  |  |  |  |  |
| Per 1 SD increase | 192 / 5437 | 1.43 (1.24 – 1.65) | < .001 | 1.25 (1.08 – 1.46) | .004 | 1.25 (1.07 – 1.46) | .005 | 1.25 (1.07 – 1.45) | .005 | 1.25 (1.08 – 1.46) | .004 |
| Q1 (< 32.2) | 16 / 1361 | ref |  | ref |  | ref |  | ref |  | ref |  |
| Q2 (32.2-39.2) | 31 / 1375 | 1.28 (0.70 – 2.35) | .43 | 1.24 (0.67 – 2.29) | .50 | 1.23 (0.66 – 2.28) | .51 | 1.25 (0.67 – 2.31) | .48 | 1.24 (0.67 – 2.29) | .50 |
| Q3 (39.3-46) | 55 / 1347 | 2.19 (1.25 – 3.84) | .006 | 1.97 (1.11 – 3.49) | .021 | 1.96 (1.11 – 3.48) | .021 | 1.93 (1.09 – 3.43) | .025 | 1.96 (1.11 – 3.48) | .021 |
| Q4 (≥ 46.1) | 90 / 1354 | 2.97 (1.73 – 5.12) | < .001 | 2.26 (1.28 – 3.96) | .005 | 2.24 (1.27 – 3.93) | .005 | 2.24 (1.28 – 3.94) | .005 | 2.26 (1.29 – 3.96) | .005 |
| **MMX** |  |  |  |  |  |  |  |  |  |  |  |
| Per 1 SD increase | 192 / 5437 | 1.20 (1.03 – 1.39) | .017 | 1.12 (0.96 – 1.31) | .16 | 1.12 (0.96 – 1.31) | .15 | 1.12 (0.95 – 1.31) | .18 | 1.12 (0.96 – 1.32) | .14 |
| Q1 (< 49.9) | 31 / 1391 | ref |  | ref |  | ref |  | ref |  | ref |  |
| Q2 (49.9-53.7) | 42 / 1355 | 1.12 (0.70 – 1.78) | .64 | 1.12 (0.70 – 1.80) | .63 | 1.12 (0.70 – 1.79) | .65 | 1.13 (0.71 – 1.81) | .61 | 1.12 (0.70 – 1.79) | .65 |
| Q3 (53.8-58.1) | 58 / 1338 | 1.48 (0.95 – 2.31) | .08 | 1.39 (0.89 – 2.17) | .15 | 1.39 (0.89 – 2.17) | .15 | 1.34 (0.86 – 2.11) | .20 | 1.38 (0.88 – 2.16) | .16 |
| Q4 (≥ 58.2) | 61 / 1353 | 1.46 (0.92 – 2.30) | .11 | 1.28 (0.79 – 2.06) | .31 | 1.28 (0.79 – 2.06) | .31 | 1.26 (0.78 – 2.04) | .34 | 1.28 (0.79 – 2.06) | .31 |

CI, confidence interval; CVD, cardiovascular disease; HR, hazard ratio; IVX, inflammation vulnerability index; MMX, metabolic malnutrition index; MVX, metabolic vulnerability index; Q, quartile; SD, standard deviation

Model 1: Age and sex
Model 2: Model 1 plus smoking status, history of type 2 diabetes, systolic blood pressure, total cholesterol, high-density lipoprotein cholesterol, triglycerides, BMI, estimated GFR, alcohol intake, antihypertensive medication use, history of cardiovascular disease, and TPOAb positivity
Model 3: Model 2 plus FT3

Model 4: Model 2 plus FT4

Model 5: Model 2 plus TSH

**Supplemental Material 6.** Associations of MVX, IVX and MMX with non-CVD mortality

|  |  | **Model 1** |  | **Model 2** |  | **Model 3** |  | **Model 4** |  | **Model 5** |  |
| --- | --- | --- | --- | --- | --- | --- | --- | --- | --- | --- | --- |
| **Exposures** | **Events/**  **Total** | **HR (95% CI)** | ***p-*value** | **HR (95% CI)** | ***p-*value** | **HR (95% CI)** | ***p-*value** | **HR (95% CI)** | ***p-*value** | **HR (95% CI)** | ***p-*value** |
| **MVX** |  |  |  |  |  |  |  |  |  |  |  |
| Per 1 SD increase | 614 / 5437 | 1.36 (1.24 – 1.48) | < .001 | 1.25 (1.14 – 1.38) | < .001 | 1.26 (1.15 – 1.38) | < .001 | 1.25 (1.14 – 1.37) | < .001 | 1.25 (1.14 – 1.38) | < .001 |
| Q1 (< 39) | 87 / 1373 | ref |  | ref |  | ref |  | ref |  | ref |  |
| Q2 (39-45) | 122 / 1348 | 1.27 (0.96 – 1.67) | 0.093 | 1.17 (0.89 – 1.55) | .26 | 1.17 (0.89 – 1.55) | .26 | 1.17 (0.89 – 1.55) | .26 | 1.17 (0.89 – 1.55) | .26 |
| Q3 (45.1-50.9) | 156 / 1362 | 1.41 (1.08 – 1.85) | .011 | 1.21 (0.92 – 1.59) | .17 | 1.22 (0.93 – 1.60) | .16 | 1.21 (0.92 – 1.59) | .17 | 1.21 (0.92 – 1.59) | .17 |
| Q4 (≥ 51) | 249 / 1354 | 2.23 (1.72 – 2.89) | < .001 | 1.83 (1.40 – 2.39) | < .001 | 1.84 (1.40 – 2.40) | < .001 | 1.82 (1.39 – 2.38) | < .001 | 1.83 (1.40 – 2.39) | < .001 |
| **IVX** |  |  |  |  |  |  |  |  |  |  |  |
| Per 1 SD increase | 614 / 5437 | 1.31 (1.21 – 1.42) | < .001 | 1.21 (1.11 – 1.32) | < .001 | 1.21 (1.11 – 1.32) | < .001 | 1.21 (1.11 – 1.32) | < .001 | 1.21 (1.11 – 1.32) | < .001 |
| Q1 (< 32.2) | 88 / 1361 | ref |  | ref |  | ref |  | ref |  | ref |  |
| Q2 (32.2-39.2) | 129 / 1375 | 1.18 (0.90 – 1.56) | .23 | 1.09 (0.83 – 1.44) | .52 | 1.10 (0.83 – 1.45) | .52 | 1.10 (0.83 – 1.45) | .52 | 1.09 (0.83 – 1.44) | .52 |
| Q3 (39.3-46) | 154 / 1347 | 1.38 (1.06 – 1.80) | .018 | 1.18 (0.90 – 1.55) | .24 | 1.18 (0.90 – 1.55) | .23 | 1.18 (0.90 – 1.54) | .24 | 1.18 (0.90 – 1.55) | .24 |
| Q4 (≥ 46.1) | 243 / 1354 | 1.98 (1.54 – 2.55) | < .001 | 1.59 (1.22 – 2.07) | .001 | 1.60 (1.23 – 2.08) | < .001 | 1.59 (1.22 – 2.07) | .001 | 1.59 (1.22 – 2.07) | .001 |
| **MMX** |  |  |  |  |  |  |  |  |  |  |  |
| Per 1 SD increase | 614 / 5437 | 1.16 (1.07 – 1.27) | < .001 | 1.15 (1.05 – 1.26) | .002 | 1.15 (1.05 – 1.26) | .002 | 1.15 (1.05 – 1.26) | .002 | 1.15 (1.05 – 1.26) | .002 |
| Q1 (< 49.9) | 125 / 1391 | ref |  | ref |  | ref |  | ref |  | ref |  |
| Q2 (49.9-53.7) | 138 / 1355 | 1.03 (0.81 – 1.32) | .81 | 1.04 (0.81 – 1.33) | .77 | 1.04 (0.81 – 1.33) | .77 | 1.04 (0.81 – 1.33) | .76 | 1.04 (0.81 – 1.33) | .77 |
| Q3 (53.8-58.1) | 152 / 1338 | 1.12 (0.88 – 1.43) | .34 | 1.10 (0.86 – 1.41) | .44 | 1.10 (0.86 – 1.41) | .44 | 1.10 (0.86 – 1.40) | .46 | 1.10 (0.86 – 1.41) | .44 |
| Q4 (≥ 58.2) | 199 / 1353 | 1.46 (1.15 – 1.86) | .002 | 1.44 (1.12 – 1.85) | .004 | 1.44 (1.12 – 1.85) | .004 | 1.44 (1.12 – 1.85) | .005 | 1.44 (1.12 – 1.85) | .004 |

CI, confidence interval; CVD, cardiovascular disease; HR, hazard ratio; IVX, inflammation vulnerability index; MMX, metabolic malnutrition index; MVX, metabolic vulnerability index; Q, quartile; SD, standard deviation

Model 1: Age and sex
Model 2: Model 1 plus smoking status, history of type 2 diabetes, systolic blood pressure, total cholesterol, high-density lipoprotein cholesterol, triglycerides, BMI, estimated GFR, alcohol intake, antihypertensive medication use, history of cardiovascular disease, and TPOAb positivity
Model 3: Model 2 plus FT3

Model 4: Model 2 plus FT4

Model 5: Model 2 plus TSH
